# Supplementary material for: Using deep mutational scanning to benchmark variant effect predictors and identify disease mutations
Source: Mol Syst Biol. 2020 Jul 6;16(7):e9380. doi: 10.15252/msb.20199380 (PMC7336272; doi:10.15252/msb.20199380)
Supplement: Supplementary file 1 — Expanded View Figures PDF [file MSB-16-e9380-s001.pdf]

Expanded View Figures

Figure EV1. Correlations between computational variant effect predictors and deep mutational scanning measurements using Kendall's Tau.

A,B Same as Fig 2, but using Kendall's Tau instead of Spearman's correlation. This analysis is split into (A) human and (B) non-human proteins.

© 2020 The Authors

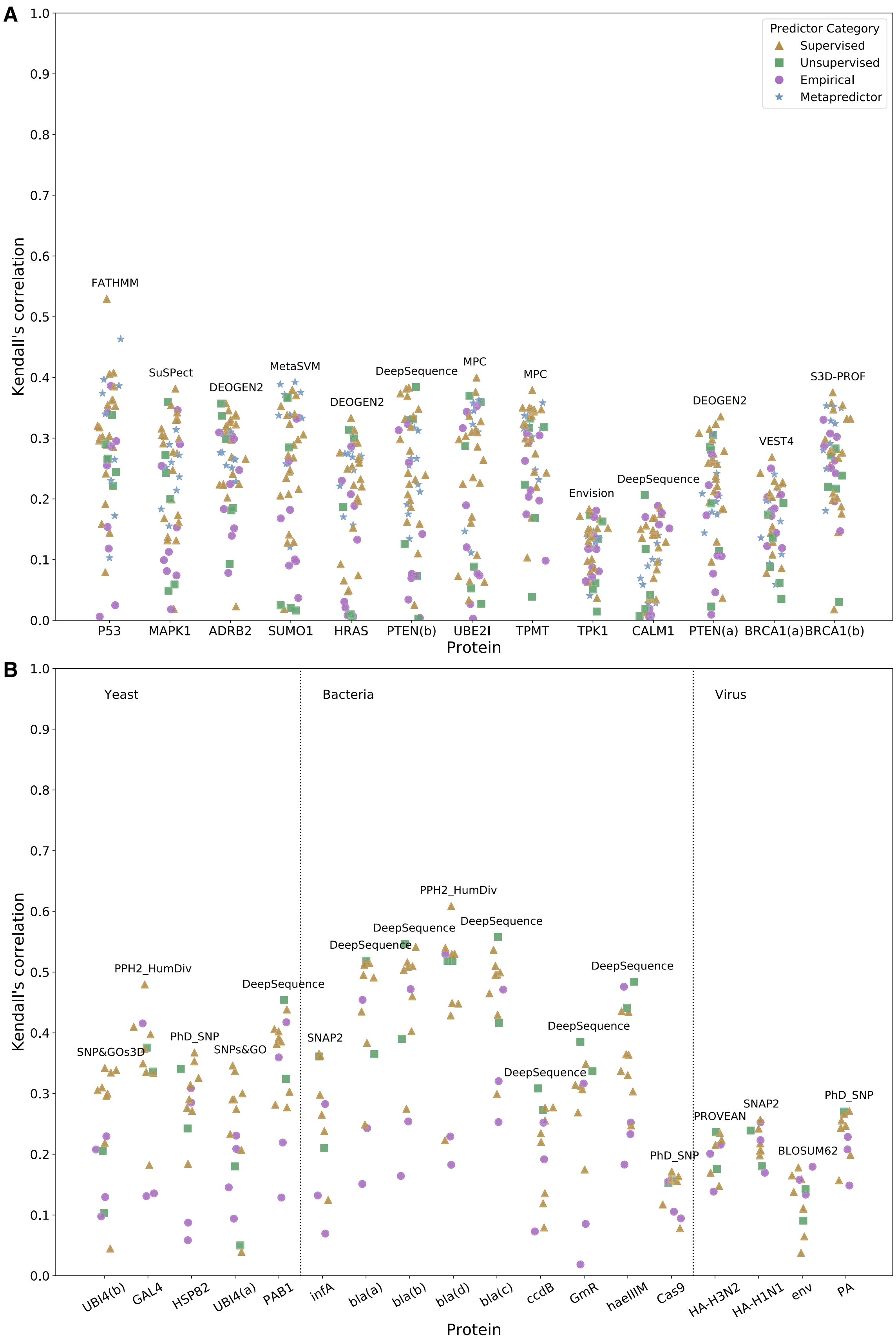

Figure EV1.

 © 2020 The Authors

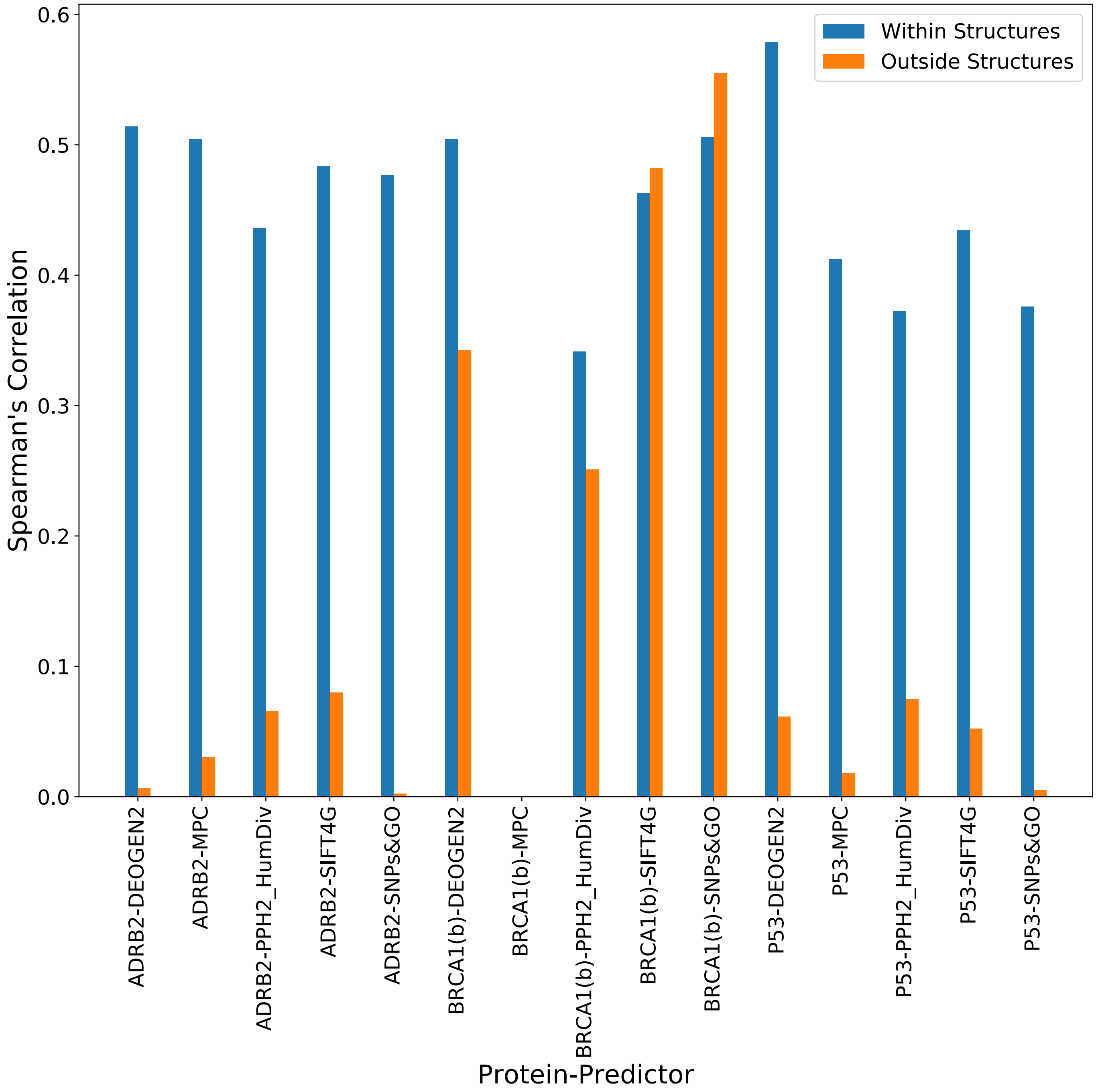

Figure EV2. Spearman's correlation between deep mutational scanning (DMS) results and variant effect predictions within and outside of areas covered by protein structures.

We assess ADRB2 (77% DMS coverage), BRCA1(b) (64% DMS coverage) and P53 (68% DMS coverage) using three predictors that incorporate some structural information (DEOGEN2, MPC and PolyPhen2_HumDiv) and predictors that do not use structures (SIFT4G and SNPs&GO). This figure shows that, while predictions tend to be much better for regions covered by known protein structures, a similar trend is observed for predictors that do and do not use structures, suggesting that the inclusion of structural information does not markedly improve the performance of VEPs.

© 2020 The Authors

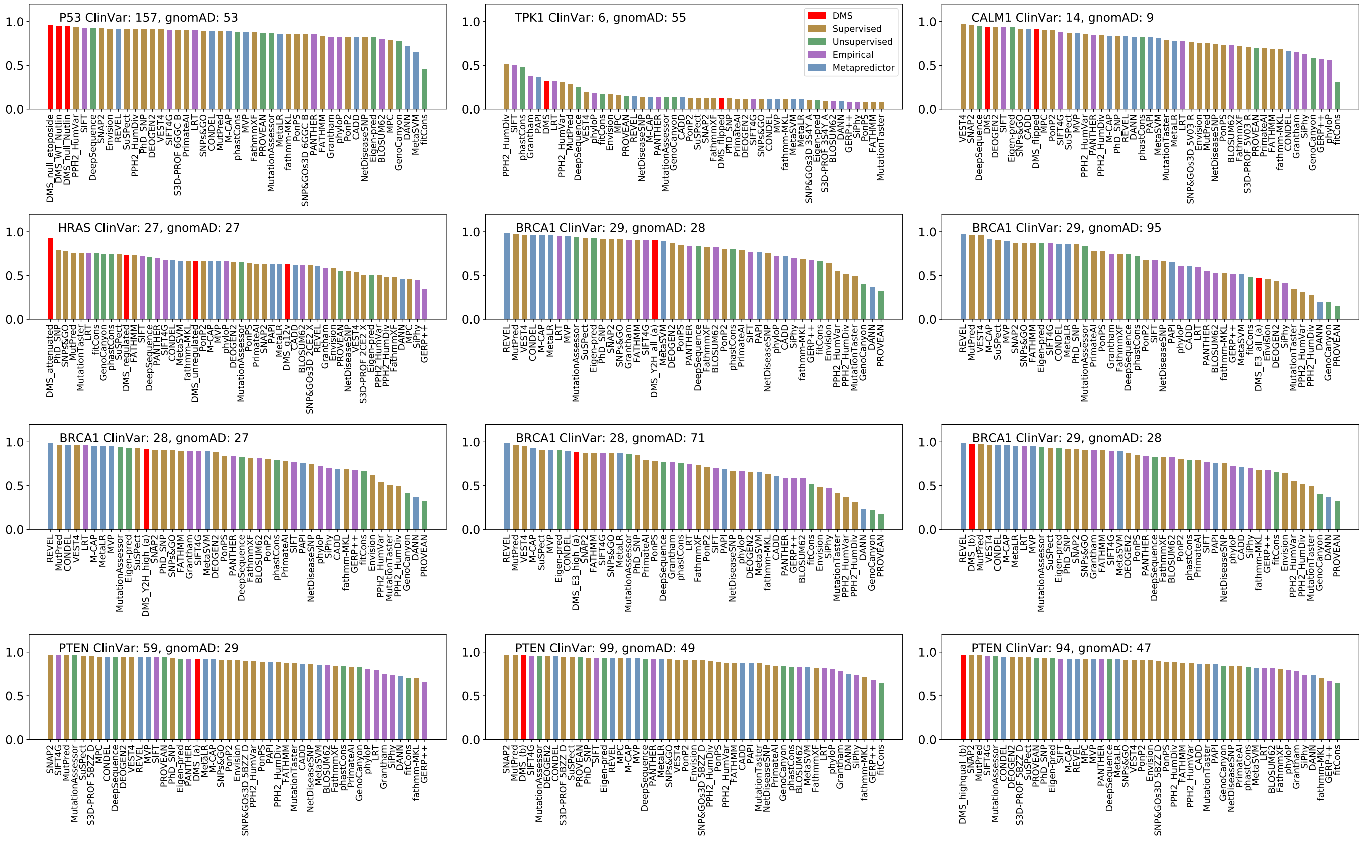

Figure EV3. Assessment of the identification of pathogenic missense variants by deep mutational scanning (DMS) data sets and variant effect predictors (VEPs) using precision–recall curves.

Precision–recall AUCs for DMS data sets and VEP predictions distinguishing between pathogenic missense variants from ClinVar and putatively benign missense variants from gnomAD. Separate plots have been drawn for DMS assays where they do not cover the same variants to maintain class balance. The sizes of both classes are indicated on the plot. The different DMS data sets for each protein are described in Table EV11.

© 2020 The Authors

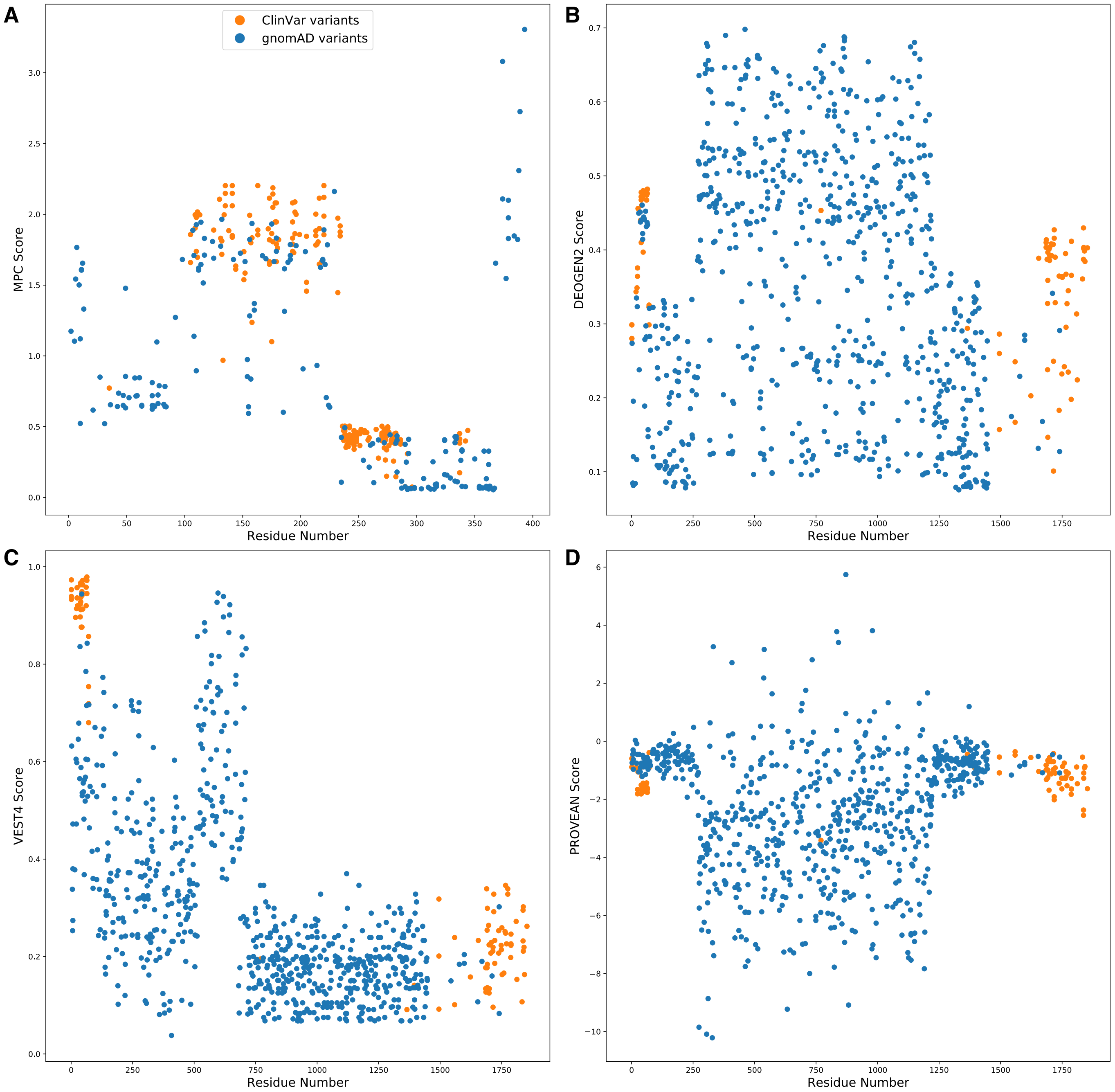

Figure EV4. Domain-specific effects observed in certain variant effect predictors (VEPs).

The distribution of predictions made by VEPs that demonstrated different domain weightings within a single protein for ClinVar and gnomAD variants.

A MPC scores for P53 variants.

B DEOGEN2 scores for BRCA1 variants.

C VEST4 scores for BRCA1 variants.

D PROVEAN scores for BRCA1 variants.

© 2020 The Authors
