# Supplementary material for: Using deep mutational scanning to benchmark variant effect predictors and identify disease mutations
Source: Mol Syst Biol. 2020 Jul 6;16(7):e9380. doi: 10.15252/msb.20199380 (PMC7336272; doi:10.15252/msb.20199380)
Supplement: Supplementary file 2 — Table EV1 [file MSB-16-e9380-s002.docx]

**Table EV1.** Summary of the computational VEPs usedd in this analysis, along with a description of utilised predictive features and the total number of DMS datasets each method generated predictions for in the format human/yeast/bacterial/viral.

| **Predictor** | **Category** | **Features** | **Data Source, method source or online predictor.** | **Total prediction sets**  **(h/y/b/v)** | **Reference** |
| --- | --- | --- | --- | --- | --- |
| DEOGEN2 | Supervised | PROVEAN, sequence conservation, pathway features, early folding predictions, interface annotations from 3D structures. | https://deogen2.mutaframe.com/ | 13/0/0/0 | (Raimondi *et al*, 2017) |
| Envision | Supervised^1^ | DMS measurements. | https://envision.gs.washington.edu/shiny/envision_new/ | 13/3/0/0 | (Gray *et al*, 2018) |
| FATHMM (weighted) | Supervised | HMM alignments, per-domain mutation consequences. | http://fathmm.biocompute.org.uk/inherited.html | 13/0/0/0 | (Shihab *et al*, 2013) |
| Fathmm-MKL | Supervised | Sequence conservation, epigenetic features, genome site features, DNA footprints. | dbNSFP database | 13/0/0/0 | (Shihab *et al*, 2015) |
| FathmmXF | Supervised | Sequence conservation, residue features, gene expression, RNA interactions, segmentation features. | http://fathmm.biocompute.org.uk/fathmm-xf/ | 13/0/0/0 | (Rogers *et al*, 2018) |
| MPC | Supervised | PolyPhen2, ‘missense badness’, sequence conservation. | dbNSFP database | 11/0/0/0 | (Samocha *et al*, 2017) |
| MutationTaster | Supervised | Regulatory features, PhyloP, phastCons, splice sites, sequence conservation, functional and domain annotations. | dbNSFP database | 13/0/0/0 | (Schwarz *et al*, 2014) |
| MutPred | Supervised | Sequence conservation, biophysical features, and sequence-based features. | dbNSFP database | 13/0/0/0 | (Pejaver *et al*, 2017) |
| NetDiseaseSNP | Supervised | Predicted structural features, SIFT, sequence conservation. | http://www.cbs.dtu.dk/services/NetDiseaseSNP/ | 13/5/9/4 | (Johansen *et al*, 2013) |
| PhD_SNP | Supervised | Sequence conservation, sequence features. | http://snps.biofold.org/snps-and-go/snps-and-go.html | 13/5/9/4 | (Capriotti *et al*, 2006) |
| PolyPhen2 (HumDiv) | Supervised | Sequence conservation, sequence features, residue-level structural features. | http://genetics.bwh.harvard.edu/pph2/ | 13/5/9/2 | (Adzhubei *et al*, 2010) |
| PolyPhen2 (HumVar) | Supervised | Sequence conservation, sequence features, residue-level structural features. | http://genetics.bwh.harvard.edu/pph2/ | 13/0/0/0 | (Adzhubei *et al*, 2010) |
| PonPS | Supervised | PonP2, sequence conservation, biophysical features, sequence features, co-evolution, predicted stability. | http://structure.bmc.lu.se/PON-PS/ | 13/0/0/0 | (Niroula & Vihinen, 2017) |
| PonP2 | Supervised | Biophysical features, GO terms, sequence conservation. | http://structure.bmc.lu.se/PON-P2/ | 13/0/0/0 | (Niroula *et al*, 2015) |
| PrimateAI | Supervised | Sequence based features, predicted structural features. | dbNSFP database | 13/0/0/0 | (Sundaram *et al*, 2018) |
| S3D-PROF | Supervised | Structural environment, sequence conservation. | http://snps.biofold.org/snps-and-go/snps-and-go-3d.html | 12/5/7/2 | (Capriotti & Altman, 2011) |
| SNAP2 | Supervised | Biophysical features, sequence conservation, *predicted* structural features, co-evolution, residue annotations. | https://www.rostlab.org/services/snap/ | 13/5/9/4 | (Hecht *et al*, 2015) |
| SNPs&GO | Supervised | Sequence conservation, sequence features, PANTHER output, GO terms | http://snps.biofold.org/snps-and-go/snps-and-go.html | 13/5/9/4 | (Capriotti *et al*, 2013) |
| SNP&GOs3D | Supervised | PANTHER, GO terms, structural environment, sequence conservation. | http://snps.biofold.org/snps-and-go/snps-and-go-3d.html | 12/5/7/2 | (Capriotti & Altman, 2011) |
| SuSPect | Supervised | Network centrality, uniprot annotations, sequence conservation, predicted surface accessibility. | http://www.sbg.bio.ic.ac.uk/suspect/about.html | 13/5/9/4 | (Yates *et al*, 2014) |
| VEST4 | Supervised | 86 pre-calculated features from SNVBox. | https://www.cravat.us/CRAVAT/ | 13/0/0/0 | (Carter *et al*, 2013) |
| DeepSequence | Unsupervised | Sequence conservation. | https://github.com/debbiemarkslab/DeepSequence | 13/5/9/3 | (Riesselman *et al*, 2018) |
| fitCons | Unsupervised | DNA accessibility, transcription, epigenetic features. | dbNSFP database | 13/0/0/0 | (Gulko *et al*, 2015) |
| GenoCanyon | Unsupervised | Sequence conservation, biochemical signal. | dbNSFP database | 13/0/0/0 | (Lu *et al*, 2015) |
| phastCons | Unsupervised | Sequence conservation, | dbNSFP database | 13/0/0/0 | (Siepel & Haussler, 2005) |
| PROVEAN | Unsupervised | Sequence conservation. | http://provean.jcvi.org/index.php | 13/5/9/4 | (Choi *et al*, 2012) |
| MutationAssessor | Unsupervised | Sequence conservation, conservation between subfamilies. | dbNSFP database | 12/0/0/0 | (Reva *et al*, 2011) |
| BLOSUM62 | Empirical (substitution matrix) | Sequence conservation. | https://www.ncbi.nlm.nih.gov/Class/FieldGuide/BLOSUM62.txt | 13/5/9/4 | (Henikoff & Henikoff, 1992) |
| GERP++ | Empirical | Sequence conservation. | dbNSFP database | 13/0/0/0 | (Davydov *et al*, 2010) |
| Grantham | Empirical (substitution matrix) | Amino acid property differences. | Matrix available in reference. | 13/5/9/4 | (Grantham, 1974) |
| LRT | Empirical | Sequence conservation. | dbNSFP database | 13/0/0/0 | (Chun & Fay, 2009) |
| PANTHER | Unsupervised | Alignment scores from protein subfamilies. | http://snps.biofold.org/snps-and-go/snps-and-go.html | 13/4/1/0 | (Thomas & Kejariwal) |
| phyloP | Empirical | Sequence conservation. | http://papi.unipv.it/ | 13/0/0/0 | (Pollard *et al*, 2010) |
| SIFT | Empirical | Sequence conservation. | https://sift.bii.a-star.edu.sg/www/code.html | 13/5/9/4 | (Sim *et al*, 2012) |
| SIFT4G | Empirical | Sequence conservation. | dbNSFP database | 13/0/0/0 | (Vaser *et al*, 2016) |
| SiPhy | Empirical | Sequence conservation. | dbNSFP database | 13/0/0/0 | (Garber *et al*, 2009) |
| CADD | Metapredictor | 949 features including numerous other predictors. Uses the same feature set as DANN. | https://cadd.gs.washington.edu/snv | 13/0/0/0 | (Kircher *et al*, 2014) |
| CONDEL | Metapredictor | Mutation Assessor, SIFT, PolyPhen2, MAPP, Pfam E-value, Fathmm. | http://bbglab.irbbarcelona.org/fannsdb/ | 13/0/0/0 | (González-Pérez & López-Bigas, 2011) |
| DANN | Metapredictor | 949 features including numerous other predictors. Uses the same feature set as CADD. | dbNSFP database | 13/0/0/0 | (Quang *et al*, 2015) |
| Eigen | Metapredictor^2^ | SIFT, PolyPhen2, MutationAssessor, conservation metrics. | dbNSFP database | 13/0/0/0 | (Ionita-Laza *et al*, 2016) |
| M-CAP | Metapredictor | SIFT, PolyPhen2, CADD, MetaLR, MutationTaster, MutationAssessor, Fathmm, LRT, evolutionary conservation metrics, substitution matrices. | http://bejerano.stanford.edu/mcap/ | 13/0/0/0 | (Jagadeesh *et al*, 2016) |
| MetaLR | Metapredictor | SIFT, PolyPhen2, GERP++, MutationTaster, MutationAssessor, Fathmm, LRT, SiPhy, PhyloP. | dbNSFP database | 13/0/0/0 | (Dong *et al*, 2015) |
| MetaSVM | Metapredictor | SIFT, PolyPhen2, GERP++, MutationTaster, MutationAssessor, Fathmm, LRT, SiPhy, PhyloP. | dbNSFP database | 13/0/0/0 | (Dong *et al*, 2015) |
| MVP | Metapredictor | Sequence context, sequence conservation, conservation metrics, predicted structural features, mutational tolerance, Eigen, VEST3, MutationTaster, PolyPhen2, SIFT, PROVEAN, fathmm-MKL, FATHMM, MutationAssessor, LRT. | dbNSFP database | 13/0/0/0 | (Qi *et al*, 2018) |
| PAPI | Metapredictor | PolyPhen2, SIFT, PseAA RF model (evolutionary conservation metrics, sequence environment). | http://papi.unipv.it/ | 13/0/0/0 | (Limongelli *et al*, 2015) |
| REVEL | Metapredictor | MutPred, PROVEAN, SIFT, PolyPhen2, LRT, MutationTaster, MutationAssessor, Fathmm, VEST3, GERP++, SiPhy, PhyloP. | https://sites.google.com/site/revelgenomics/ | 13/0/0/0 | (Ioannidis *et al*, 2016) |

^1^ Envision is a supervised method, but trained on DMS data, thus not used for calculating the RankScore in table EV7.

^2^ Unlike the other metapredictors, Eigen uses an unsupervised machine learning method.

**References**

Capriotti E, Calabrese R, Casadio R (2006) Predicting the insurgence of human genetic diseases associated to single point protein mutations with

support vector machines and evolutionary information. Bioinformatics 22: 2729 – 2734

Choi Y, Sims GE, Murphy S, Miller JR, Chan AP (2012) Predicting the functional effect of amino acid substitutions and indels. PLoS ONE 7: e46688

Chun S, Fay JC (2009) Identification of deleterious mutations within three human genomes. Genome Res 19: 1553 – 1561

Dong C, Wei P, Jian X, Gibbs R, Boerwinkle E, Wang K, Liu X (2015) Comparison and integration of deleteriousness prediction methods for

nonsynonymous SNVs in whole exome sequencing studies. Hum Mol Genet 24: 2125 – 2137

González-Pérez A, López-Bigas N (2011) Improving the assessment of the outcome of nonsynonymous SNVs with a consensus deleteriousness score,

Condel. Am J Hum Genet 88: 440 – 449

Jagadeesh KA, Wenger AM, Berger MJ, Guturu H, Stenson PD, Cooper DN, Bernstein JA, Bejerano G (2016) M-CAP eliminates a majority of variants of

uncertain significance in clinical exomes at high sensitivity. Nat Genet 48: 1581 – 1586

Johansen MB, Izarzugaza JMG, Brunak S, Petersen TN, Gupta R (2013) Prediction of disease causing non-synonymous SNPs by the artificial

neural network predictor NetDiseaseSNP. PLoS ONE 8: e68370

Kircher M, Witten DM, Jain P, O’Roak BJ, Cooper GM, Shendure J (2014) A general framework for estimating the relative pathogenicity of human

genetic variants. Nat Genet 46: 310 – 315

Limongelli I, Marini S, Bellazzi R (2015) PaPI: pseudo amino acid composition to score human protein-coding variants. BMC Bioinformatics 16: 123

Lu Q, Hu Y, Sun J, Cheng Y, Cheung K-H, Zhao H (2015) A statistical framework to predict functional non-coding regions in the human

genome through integrated analysis of annotation data. Sci Rep 5: 1 – 13

Niroula A, Urolagin S, Vihinen M (2015) PON-P2: prediction method for fast and reliable identification of harmful variants. PLoS ONE 10: e0117380

Niroula A, Vihinen M (2017) Predicting severity of disease-causing variants. Hum Mutat 38: 357 – 364

Pejaver V, Urresti J, Lugo-Martinez J, Pagel KA, Lin GN, Nam H-J, Mort M, Cooper DN, Sebat J, Iakoucheva LM et al (2017) MutPred2: inferring the

molecular and phenotypic impact of amino acid variants. bioRxiv <https://d>oi.org/10.1101/134981 [PREPRINT]

Pollard KS, Hubisz MJ, Rosenbloom KR, Siepel A (2010) Detection of nonneutral substitution rates on mammalian phylogenies. Genome Res 20:

110 – 121

Qi H, Chen C, Zhang H, Long JJ, Chung WK, Guan Y, Shen Y (2018) MVP: predicting pathogenicity of missense variants by deep learning. bioRxiv

https://doi.org/10.1101/259390 [PREPRINT]

Quang D, Chen Y, Xie X (2015) DANN: a deep learning approach for annotating the pathogenicity of genetic variants. Bioinformatics 31:

761 – 763

Reva B, Antipin Y, Sander C (2011) Predicting the functional impact of protein mutations: application to cancer genomics. Nucleic Acids Res 39: e118

Rogers MF, Shihab HA, Mort M, Cooper DN, Gaunt TR, Campbell C (2018) FATHMM-XF: accurate prediction of pathogenic point mutations via

extended features. Bioinformatics 34: 511 – 513

Samocha KE, Kosmicki JA, Karczewski KJ, O’Donnell-Luria AH, Pierce-Hoffman E, MacArthur DG, Neale BM, Daly MJ (2017) Regional missense constraint

improves variant deleteriousness prediction. bioRxiv <https://doi.org/10>.101/148353 [PREPRINT]

Schwarz JM, Cooper DN, Schuelke M, Seelow D (2014) MutationTaster2: mutation prediction for the deep-sequencing age. Nat Methods 11: 361 – 362

Shihab HA, Rogers MF, Gough J, Mort M, Cooper DN, Day INM, Gaunt TR, Campbell C (2015) An integrative approach to predicting the functional effects

of non-coding and coding sequence variation. Bioinformatics 31: 1536 – 1543

Sundaram L, Gao H, Padigepati SR, McRae JF, Li Y, Kosmicki JA, Fritzilas N, Hakenberg J, Dutta A, Shon J et al (2018) Predicting the clinical impact

of human mutation with deep neural networks. Nat Genet 50: 1161 – 1170

Thomas PD, Kejariwal A (2004) Coding single-nucleotide polymorphisms associated with complex vs. Mendelian disease: evolutionary evidence for

differences in molecular effects. Proc Natl Acad Sci USA 101: 15398 – 15403
