# Supplementary material for: Using deep mutational scanning to benchmark variant effect predictors and identify disease mutations
Source: Mol Syst Biol. 2020 Jul 6;16(7):e9380. doi: 10.15252/msb.20199380 (PMC7336272; doi:10.15252/msb.20199380)
Supplement: Supplementary file 3 — Table EV2 [file MSB-16-e9380-s003.docx]

**Table EV2.** Summary of the DMS datasets used in this analysis, including functional assay and method of mutagenesis. We also note the specific DMS assay from each study we use for calculating correlation with the VEP predictions.

| **Protein(s) (Uniprot ID)** | **Organism** | **Functional Assay** | **Mutagenesis Method** | **Utilised assay** | **Access date** | **Reference** |
| --- | --- | --- | --- | --- | --- | --- |
| UBE2I (P63279)  SUMO1 (P63165)  TPK1 (Q9H3S3)  CALM1 (P0DP23) | Human | POPCode, a variant of multiple-site directed mutagenesis. | Competitive growth assay in yeast. | (screen.score column)  UBE2I_flipped_scores  SUMO1_flipped_scores  TPK1_flipped_scores  CALM1_flipped_scores | 12/10/2018 | (Weile *et al*, 2017) |
| BRCA1(a) (P38398) | Human | Systematic site-directed mutagenesis. | Yeast two-hybrid assay and phage display. | E3_score_800_filter_pass | 12/10/2018 | (Starita *et al*, 2015) |
| BRCA1(b) (P38398) | Human | Systematic site-directed mutagenesis. | Competitive growth assay in HAP1 cells. | function.score.mean | 14/02/2020 | (Findlay *et al*, 2018) |
| P53 (P04637) | Human | Systematic site-directed mutagenesis. | Competitive growth assay in the presence of P53 agonists | A549_p53WT_Nutlin-3_Z-score | 12/10/2018 | (Giacomelli *et al*, 2018) |
| HRas (P01112) | Human | Systematic site-directed mutagenesis. | Two-hybrid assay. | Ras-G12V | 12/10/2018 | (Bandaru *et al*, 2017) |
| MAPK1 (P28482) | Human | Systematic site-directed mutagenesis. | Competitive growth assay. | Doxycycline | 12/10/2018 | (Brenan *et al*, 2016) |
| PTEN(a) (P60484)  TPMT (P51580) | Human | Systematic site-directed mutagenesis. | Fluorescence of a GFP fusion protein. | score  score | 12/10/2018 | (Matreyek *et al*, 2018) |
| PTEN(b) (P60484) | Human | Systematic site-directed mutagenesis. | Disruption of an artificial genetic circuit in yeast. | Cum_score_high_conf | 14/02/2020 | (Mighell *et al*, 2018) |
| ADRB2 (P07550) | Human | Systematic site-directed mutagenesis. | Pathway-specific reporter  gene transcription  assessed by RNA-seq. | condition_0.625 | 31/04/2019 | (Jones *et al*, 2019) |
| HSP82 (P02829) | Yeast | Systematic site-directed mutagenesis. | Competitive growth assay. | norm_ratiochange | 12/10/2018 | (Mishra *et al*, 2016) |
| UBI4(a) (P0CG63) | Yeast | Systematic site-directed mutagenesis. | Competitive growth assay. | selection_coefficient | 12/10/2018 | (Roscoe *et al*, 2013) |
| UBI4(b) (P0CG63) | Yeast | Site directed mutagenesis by cassette ligation. | Fluorescence activated cell sorting (FACS). | Relative_E1-activity_limiting | 12/10/2018 | (Roscoe & Bolon, 2014) |
| PAB1 (P04147) | Yeast | Random mutagenesis by error-prone PCR. | Competitive growth assay. | Linear | 12/10/2018 | (Melamed *et al*, 2013) |
| GAL4 (P04386) | Yeast | Systematic site-directed mutagenesis. | Two-hybrid assay. | SEL_A_24h | 12/10/2018 | (Kitzman *et al*, 2015) |
| infA (P69222) | E. coli | Systematic site-directed mutagenesis. | Competitive growth assay. | fitness_min | 12/10/2018 | (Kelsic *et al*, 2016) |
| GmR (N/A)^1^ | E. coli | Systematic site-directed mutagenesis. | Antibiotic resistance. | 37C | 12/10/2018 | (Dandage *et al*, 2018) |
| bla(a) (P62593) | E. coli | Systematic site-directed mutagenesis. | Antibiotic resistance. | Fitness | 12/10/2018 | (Firnberg *et al*, 2014) |
| bla(b) (P62593) | E. coli | Systematic site-directed mutagenesis. | Antibiotic resistance. | Ampicillin_2500 | 12/10/2018 | (Stiffler *et al*, 2015) |
| bla(c) (P62593) | E. coli | Random mutagenesis. | Antibiotic resistance. | MIC_Score_WT | 12/10/2018 | (Jacquier *et al*, 2013) |
| bla(d) (P62593) | E. coli | Random and site-directed mutagenesis. | Antibiotic resistance. | DMS | 12/10/2018 | (Deng *et al*, 2012) |
| ccdB (P62554) | E. coli | Systematic site-directed mutagenesis. | Reverse survival assay (for toxin activity). | MSseq | 12/10/2018 | (Adkar *et al*, 2012) |
| haeIIIM (P20589) | H. aegyptius | Random mutagenesis. | Competitive growth assay. | W_rel_G17 | 12/10/2018 | (Rockah-Shmuel *et al*, 2015) |
| Cas9 (Q99ZW2) | S. pyrogenes | Random mutagenesis by error-prone PCR. | Survival assay. | Log2_Fold_Change_after_Positive_Selection | 12/10/2018 | (Spencer & Zhang, 2017) |
| env (P03377) | HIV virus | Systematic site-directed mutagenesis. | Competitive replication assay. | Site_preferences | 12/10/2018 | (Haddox *et al*, 2016) |
| HA-H1N1 (A0A2Z5U3Z0) | Influenza virus | Helper virus. | Competitive replication assay. | Site_preferences | 12/10/2018 | (Doud & Bloom, 2016) |
| HA-H3N2 (A0A097PF60) | Influenza virus | Helper virus. | Competitive replication assay. | avg_prefs | 12/10/2018 | (Lee *et al*, 2018) |
| PA (P15659) | Influenza virus | Helper virus. | Competitive replication assay. | RF_index | 12/10/2018 | (Wu *et al*, 2015) |

^1^ The GmR sequence used in the study does not correspond to any Uniprot ID. We used the sequence in the paper for predictor input.

**References**

Brenan L, Andreev A, Cohen O, Pantel S, Kamburov A, Cacchiarelli D, Persky NS, Zhu C, Bagul M, Goetz EM et al (2016) Phenotypic characterization of

a comprehensive set of MAPK1/ERK2 missense mutants. Cell Rep 17: 1171 – 1183

Giacomelli AO, Yang X, Lintner RE, McFarland JM, Duby M, Kim J, Howard TP, Takeda DY, Ly SH, Kim E et al (2018) Mutational processes shape the

landscape of TP53 mutations in human cancer. Nat Genet 50: 1381

Jones EM, Lubock NB, Venkatakrishnan AJ, Wang J, Tseng AM, Paggi JM, Latorraca NR, Cancilla D, Satyadi M, Davis JE et al (2019) Structural

and functional characterization of G protein-coupled receptors with deep mutational scanning. bioRxiv <https://doi.org/10.1101/623108> [PREPRINT]

Kelsic ED, Chung H, Cohen N, Park J, Wang HH, Kishony R (2016) RNA structural determinants of optimal codons revealed by MAGE-seq. Cell

18 Syst 3: 563 – 571.e6

Melamed D, Young DL, Gamble CE, Miller CR, Fields S (2013) Deep mutational scanning of an RRM domain of the Saccharomyces cerevisiae poly(A)-

binding protein. RNA 19: 1537 – 1551

Mishra P, Flynn JM, Starr TN, Bolon DNA (2016) Systematic mutant analyses elucidate general and client-specific aspects of Hsp90 function. Cell Rep

15: 588 – 598

Rockah-Shmuel L, Tóth-Petróczy Á, Tawfik DS (2015) Systematic mapping of protein mutational space by prolonged drift reveals the deleterious effects

31 of seemingly neutral mutations. PLoS Comput Biol 11: e1004421

Roscoe BP, Thayer KM, Zeldovich KB, Fushman D, Bolon DNA (2013) Analyses of the effects of all ubiquitin point mutants on yeast growth rate. J Mol

33 Biol 425: 1363 – 1377

Roscoe BP, Bolon DNA (2014) Systematic exploration of ubiquitin sequence, E1 activation efficiency, and experimental fitness in yeast. J Mol Biol 426:

34 2854 – 2870

Spencer JM, Zhang X (2017) Deep mutational scanning of S. pyogenes Cas9 reveals important functional domains. Sci Rep 7: 16836
